# *In Vitro* Evaluation of *Spirulina platensis* Aqueous Extract and C-Phycocyanin on Skin Cell Metabolic Activity, Wound Healing and Antioxidant Activities

**DOI:** 10.3390/molecules31214020

**Published:** 2026-11-08

**Authors:** Gabija Nemeikšė, Jurga Bernatonienė, Asta Špadienė, Raimondas Benetis, Daiva Galinytė, Nijolė Savickienė, Gabrielė Balčiūnaitė-Murzienė, Aistė Jekabsone, Ramunė Morkūnienė

**Affiliations:** 1 Faculty of Pharmacy, Lithuanian University of Health Sciences, LT-50162 Kaunas, Lithuania; gabija.nemeikse@lsmu.lt (G.N.); jurga.bernatoniene@lsmu.lt (J.B.); asta.spadiene@lsmu.lt (A.Š.); raimondas.benetis@lsmu.lt (R.B.); daiva.galinyte@stud.lsmu.lt (D.G.); nijole.savickiene@lsmu.lt (N.S.); gabriele.balciunaite-murziene@lsmu.lt (G.B.-M.); aiste.jekabsone@lsmu.lt (A.J.); 2 Institute of Cardiology, Lithuanian University of Health Sciences, LT-50166, Kaunas, Lithuania

**Keywords:** *Spirulina platensis* bioactive compounds, aqueous extract, phycocyanin, skin cell culture, cellular metabolic activity, antioxidant properties

## Abstract

Various *Spirulina* species and their major pigment, Cyano-phycocyanin (C-PC), have been shown to exhibit antioxidant, anti-inflammatory, and anticancer properties, and are used in various skincare products. *Spirulina platensis* is rich in nutrients, proteins, and antioxidants, with therapeutic potential for skin protection and regeneration. Aqueous *Spirulina* extracts have gained increasing attention in recent years due to their biocompatibility; however, comparative assessment of aqueous extract and C-PC responses across non-malignant and malignant skin cells remains limited. This study aimed to evaluate the effects of *S. platensis* aqueous extract and C-PC on the cellular metabolic activity and wound healing of human skin cell lines—melanoma cells, keratinocytes, and fibroblasts—using a 24 h PrestoBlue assay and time-resolved scratch assays. The results demonstrated a concentration- and cell-type-dependent response to both preparations: at selected concentrations, *S. platensis* aqueous extract and C-PC reduced melanoma cellular metabolic activity while increasing it in keratinocytes and fibroblasts. Notably, both *S. platensis* aqueous extract and C-PC significantly accelerated wound closure at selected concentrations in keratinocytes and fibroblasts but not in melanoma cell cultures. Additionally, the *S. platensis* extract exhibited strong antioxidant activity, which correlated with its phenolic and flavonoid content. In conclusion, *S. platensis* aqueous extract and C-PC exhibit concentration- and cell-type-dependent effects on skin cell metabolic activity and scratch closure, supporting further investigation of their potential for skin-repair applications.

## 1. Introduction

As the body’s outermost layer, the skin serves as the primary barrier against pathogens, environmental stressors, and transepidermal water loss while maintaining physiological homeostasis [1]. However, its function deteriorates with aging and can be further impaired by various diseases—such as diabetes, kidney, liver and autoimmune disorders—as well as by environmental factors [2,3,4]. These conditions reduce keratinocyte proliferation, diminished fibroblast function, promote chronic inflammation, and delay wound healing [1]. Consequently, interest in safe bioactive compounds that can support skin regeneration has increased.

Natural compounds derived from microalgae represent promising candidates in dermatology. *Spirulina platensis* is a multicellular filamentous cyanobacterium that naturally forms spiral-shaped colonies in warm, alkaline waters. Its biochemical profile includes proteins, essential amino acids, fatty acids, polysaccharides, vitamins and minerals. The main bioactive components—such as C-phycocyanin (C-PC), β-carotene, sulfated polysaccharides, γ-linolenic acid, and sulfolipids—contribute to its antioxidant, anti-inflammatory, antimicrobial, photoprotective, and anticancer properties [5]. Aqueous, ethanolic, methanolic or acetone extracts of *S. platensis* contain distinct groups of bioactive compounds that may affect skin cells differently. Ethanolic extracts contain higher levels of total phenolics (such as p-coumaric, chlorogenic, and caffeic acids) and flavonoids (including quercetin and related derivatives), as well as greater concentrations of carotenoids and chlorophylls [6]. Methanolic extracts contain compounds such as hexadecanoic acid, 9-octadecenoic acid, tetradecanoic acid, and phytol. Studies also report that both ethanolic and methanolic extracts contain alkaloids, glycosides, phenols, flavonoids, terpenoids, steroids, and saponins [7]. In contrast, aqueous extracts are enriched in phycobiliproteins (C-phycocyanin, allo-phycocyanin, phycoerythrin), free amino acids, carbohydrates and total protein [8].

C-PC, one of the major bioactive pigments found in *S. platensis* and other cyanobacteria, is a water-soluble blue phycobiliprotein that forms part of the phycobilisome complex and functions as a key light-harvesting molecule in photosynthesis [9]. It is widely used across industrial sectors due to its intense blue coloration and natural fluorescence. Numerous studies have demonstrated that C-PC possesses antioxidant, anti-inflammatory and anticancer activities [10,11,12].

Previous studies show that *S. platensis* crude extracts can enhance keratinocyte proliferation, with 0.05–0.1% concentrations producing the strong proliferative response likely due to its high C-PC content [13]. Keratinocytes play a central role in restoring the epidermis after injury by driving epithelialization [14]. Accordingly, topical formulations enriched with *S. platensis* aqueous crude extract (up to 1.125%) significantly enhanced wound-healing responses in keratinocyte cultures and promoted wound repair in a mouse model, demonstrating the regenerative potential of *Spirulina*-derived preparations [13,15]. Another study showed that an aqueous extract of *S. platensis* enhanced *in vitro* wound closure in human dermal fibroblasts [16]. C-PC has been shown to promote fibroblast proliferation in a dose-dependent manner, enhance the G1 phase of the cell cycle, and upregulate cyclin-dependent kinases 1 and 2 through a mechanism independent of uPA [17]. Sevimi Gur et al. compared crude *Spirulina* extract and C-PC, reporting enhanced human keratinocyte viability with no significant difference between preparations over the tested concentration range (0.0335–33.5 µg/mL), while 1.25% C-PC produced a more favorable tissue-regeneration response in a rat wound model [18]. In addition, C-PC treatment has been shown to accelerate wound healing in mouse models of *C. albicans* infection and in full-thickness dermal wounds [19]. Similarly, *S. platensis* nanophytosome gel significantly enhanced wound healing in rats by promoting re-epithelialization, increasing collagen deposition, suppressing inflammatory signaling, and down-regulating HGMB-1 protein expression [20]. Recent research has highlighted the anticancer potential of *Spirulina platensis*-derived compounds in cell culture models, with C-PC emerging as the most prominent bioactive compound responsible for these effects [10,21]. High concentrations of ethanolic and methanolic *S. platensis* extracts (≤5 mg/mL) strongly inhibited melanoma cell viability [8]. These multifunctional biological activities make *S. platensis*-derived preparations strong candidates for dermatological use, supporting their potential in both cosmetic and therapeutic applications [22,23].

Taken together, these findings suggest that *S. platensis*—and its compound C-PC itself—may help protect skin and support tissue repair following injury, infection, or surgical excision of skin tumors.

Although previous studies have demonstrated the wound-healing potential of S*pirulina* extracts and C-PC, further comparative studies are needed to assess their concentration-dependent effects across non-malignant and malignant skin cell types. Therefore, the aim is to compare the effects of *S. platensis* aqueous extracts and a commercial C-PC preparation on non-malignant human skin cells (HaCaT keratinocytes and BJ-5ta fibroblasts) and A375 melanoma cells by assessing their metabolic activity, wound-healing capacity, and the antioxidant activity of both preparations.

## 2. Results

### 2.1. Chemical Composition of the Spirulina platensis Extract

Lyophilized powder of *Spirulina platensis* (100% *Spirulina*) composition values was based on the manufacturer’s specification provided by Bulk Powders™ (Pure Series, Colchester, UK). Parameters per 100 g: protein 63.0 g, carbohydrates 20.0 g, fat 6.0 g and dietary fibers 3.6 g.

Amino acid analysis of *Spirulina platensis* extract performed using an HPLC system coupled to a Xevo TQD mass spectrometer (Waters Corporation, Milford, MA, USA) revealed a protein source rich in non-essential amino acids (Glutamate, Alanine, Serine and Glycine) and two essential amino acids, Histidine and Leucine (Table 1). The highest level was glutamic acid (116.84 μg/g dry weight (DW)), which confirms the typical result in *Spirulina* species.

The content of C-PC, a pigment–protein complex, in *Spirulina* aqueous extracts was determined using distilled water and phosphate buffer (pH 6.8) as described [24]. Distilled water was used as a simple extraction medium, whereas the phosphate buffer maintained a stable pH during extraction and analysis, potentially preserving the stability and structural integrity of C-PC. We found that *Spirulina platensis* sample incubation with phosphate buffer resulted in the release of 35.65 ± 1.46 mg/g DW C-PC, with distilled water—28.05 ± 2.08 mg/g DW C-PC.

In addition, we have investigated the presence of secondary metabolites in *Spirulina* water extracts and spectrophotometrically determined the total content of phenolic compounds (TPC)—7.89 ± 0.43 mg GAE/g DW and the total flavonoid content (TFC)—20.78 ± 0.98 mg RE/g DW. The two organic acids—citric and malic—were detected and quantified using UPLC/Xevo-TQD MS (Waters Corporation, Milford, MA, USA).

Overall, these results are in agreement with previously reported data and confirm the presence of diverse bioactive compounds, including proteins, amino acids, C-PC and secondary metabolites supporting the consistency and quality of the *Spirulina platensis* aqueous extracts.

### 2.2. The Effects of Spirulina platensis Extract and C-Phycocyanin on the Cellular Metabolic Activity of Human Skin Cell Lines

We have evaluated the effects of various concentrations of aqueous extract of *S. platensis* (25, 50, 75, 100, 125, 200, 250 and 500 µg/mL) and C-PC (100, 150, 200, 250, 300, 500 µg/mL) on the human melanoma, keratinocyte and fibroblast cell lines after 24 h incubation using the PrestoBlue fluorescence assay. PrestoBlue is a resazurin-based and sensitive cell viability reagent used to measure the metabolic activity of cells, which may correlate with the number of viable cells. The concentrations of *Spirulina platensis* extract and C-PC used in this study may reflect physiologically achievable levels of bioactive components present in aqueous *S. platensis* extracts. Therapeutic *in vitro* concentrations of C-PC typically range from 5 to 500 µg/mL, and even up to 1000 µg/mL, used to investigate strong pharmacological or cytotoxic effects.

**Figure 1:**
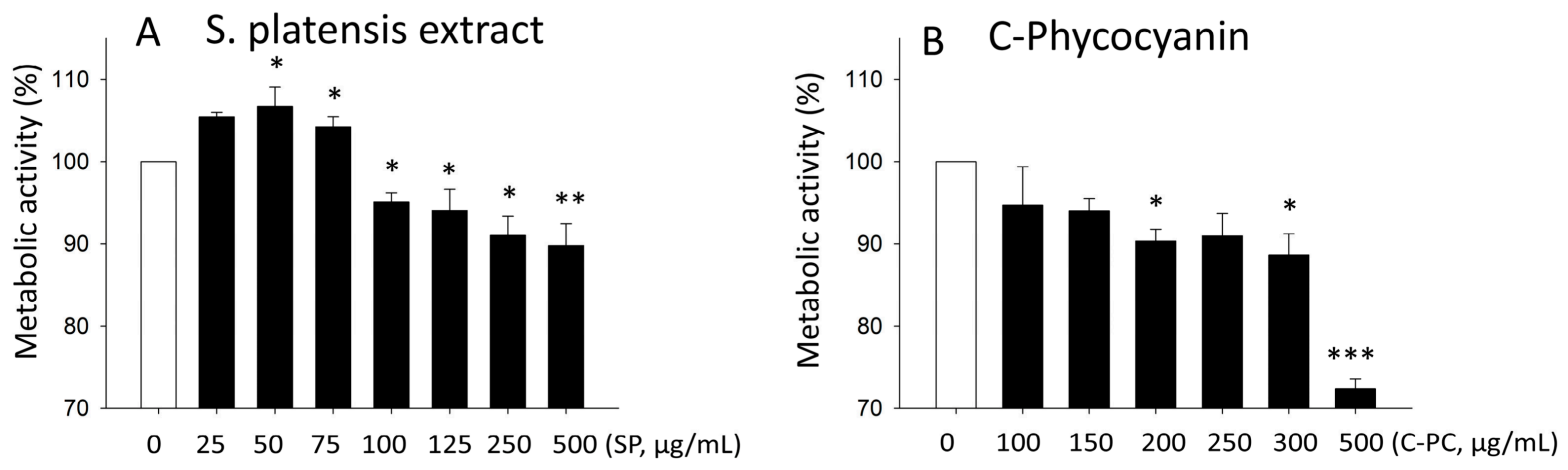
The effect of *S. platensis* aqueous extract and C-Phycocyanin on the cellular metabolic activity of melanoma cells. Cellular metabolic activity was assessed using PrestoBlue reagent. After 24 h of treatment with different concentrations of *S. platensis* extract (25, 50, 75, 100, 125, 200, 250 and 500 µg/mL) and C-PC (100, 150, 200, 250, 300, 500 µg/mL), the measurement was determined at 560 nm using the Infinite 200 Pro M Nano Plex multi-plate reader. Quantitative evaluation of cellular metabolic activity, in (**A**)—*S. platensis* extract (SP); (**B**)—C-Phycocyanin (C-PC). Data are reported as the percentage of cellular metabolic activity compared to the percentage in cells without treatment (control, set at 100%). The data are presented as mean ± standard deviation (SD) of 3–5 independent experiments. Differences between experimental groups were evaluated using one-way ANOVA followed by Tukey’s post hoc test. Statistically significant difference compared with the control: ***—*p* < 0.001, **—*p* < 0.01, *—*p* < 0.05.

Figure 1A shows that low concentrations of *S. platensis*—50 and 75 µg/mL—slightly induced melanoma cellular metabolic activity by 4–7%, if compared with the control (cells not exposed to *S. platensis* extract and whose metabolic activity level was considered 100%). However, the cellular metabolic activity of melanoma cells treated with *S. platensis* extract at concentration 100 µg/mL and above was inhibited by about 10%, compared to the control group. The fluorescence-based assessment of melanoma cell viability using Hoechst 33342/PI staining further supported the partially concentration-dependent inhibitory effect of the *S. platensis* extract. The corresponding results are provided in the Supplementary Materials, Figure S1A.

In parallel, we analyzed the effect of C-PC solutions on melanoma metabolic activity after 24 h incubation (Figure 1B). Lower concentrations (100, 150, and 250 µg/mL) of C-PC did not affect melanoma metabolic activity; however, 200 and 300 µg/mL C-PC reduced cellular metabolic activity by 10% after 24 h, compared to the control. The highest C-PC concentration—500 µg/mL—had a pronounced inhibitory effect and reduced the PrestoBlue signal by 28% after 24 h. Additionally, melanoma cell viability following treatment with the C-PC solution was evaluated using a Hoechst 33342/PI-based Muvicyte fluorescence microscopy method. The analysis revealed the strong inhibitory effect at the 500 µg/mL C-PC concentration by reducing the viability by 46% compared with the control. The corresponding results are presented in the Supplementary Materials, Figure S1B.

**Figure 2:**
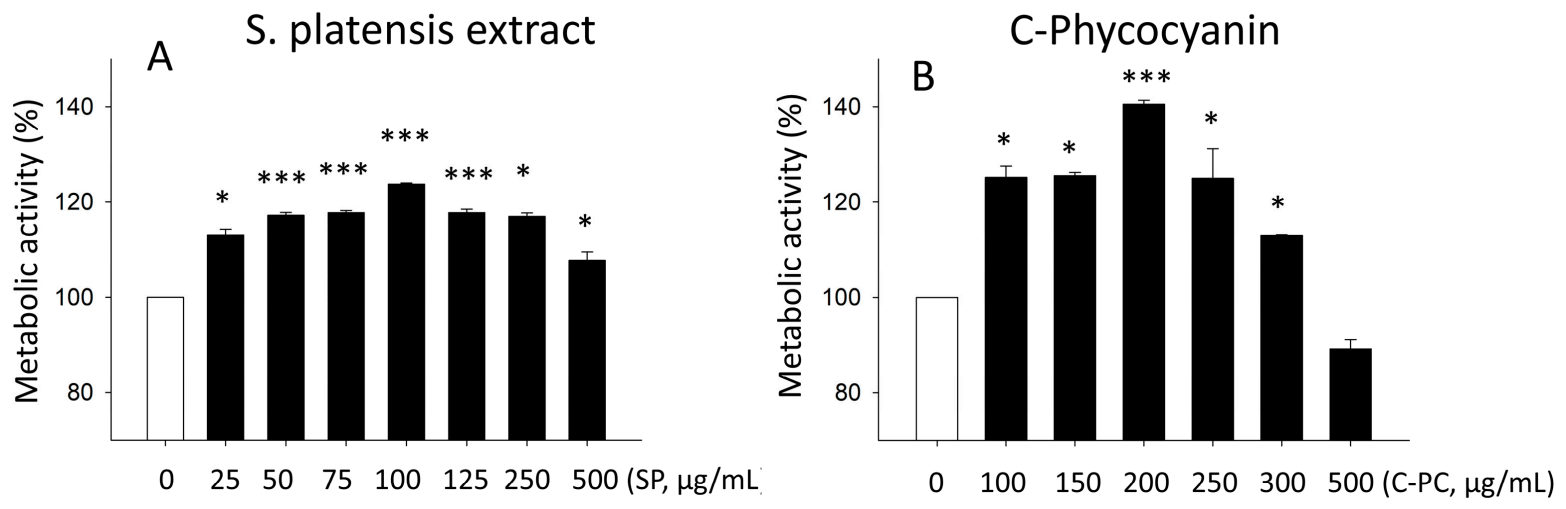
The effect of *Spirulina platensis* aqueous extract and C-Phycocyanin on the cellular metabolic activity of keratinocytes. Cellular metabolic activity was assessed using PrestoBlue reagent, as described in Figure 1. Quantitative evaluation of cellular metabolic activity, in (**A**)—*S. platensis* extract (SP); (**B**)—C-PC. The data are presented as mean ± SD of 3–5 independent experiments. Differences between experimental groups were evaluated using one-way ANOVA followed by Tukey’s post hoc test. Statistically significant differences compared with the control: ***—*p* < 0.001, *—*p* < 0.05.

Unlike melanoma cells, all concentrations of *S. platensis* extract stimulated keratinocyte cellular metabolic activity after 24 h incubation (Figure 2A), and the most effective *S. platensis* extract concentration was 100 µg/mL, which increased keratinocyte the PrestoBlue signal by 24%, compared to the control. Other *S. platensis* concentrations increased keratinocyte metabolic activity by about 10–15% after 24 h (Figure 2A).

Similarly to the effects of *S. platensis* extract, C-PC 100–300 µg/mL significantly increased keratinocyte metabolic activity—by about 20–25%—compared with the control (Figure 2B). The most powerful effect was observed with the 200 µg/mL concentration, which stimulated keratinocyte metabolic activity by up to 41%. The 500 µg/mL C-PC did not affect the keratinocyte cell culture.

**Figure 3:**
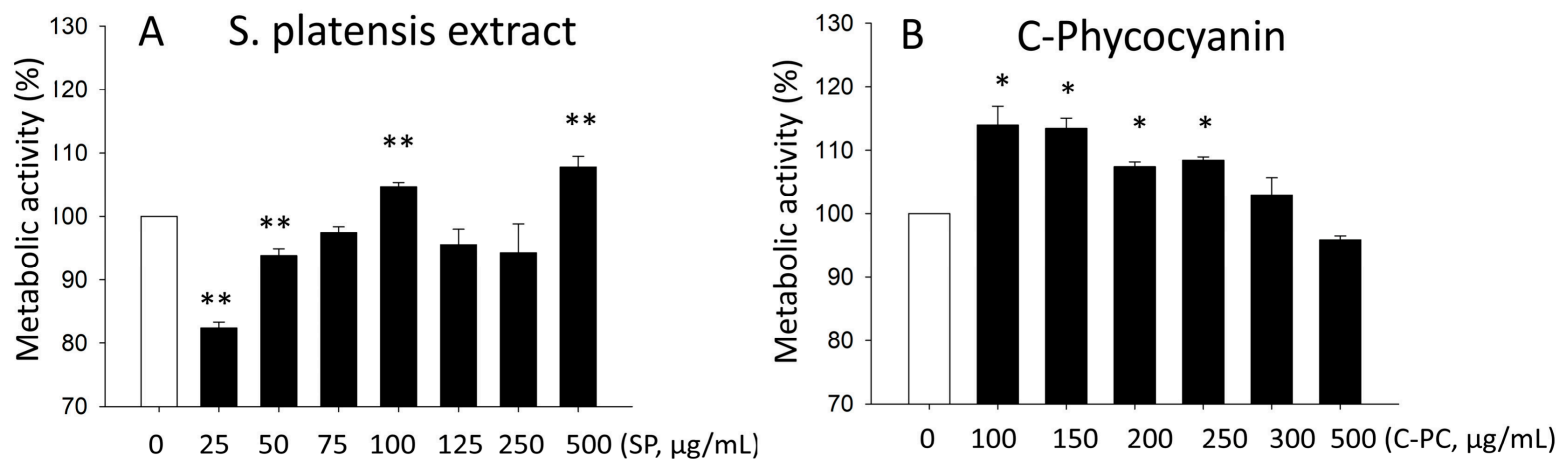
The effect of *Spirulina platensis* aqueous extract and C-Phycocyanin on the cellular metabolic activity of fibroblasts. Cellular metabolic activity was assessed using PrestoBlue reagent as described in Figure 1. Quantitative evaluation of cellular metabolic activity, in (**A**)—*S. platensis* extract (SP); (**B**)—C-PC. The data are presented as mean ± SD of 3–5 independent experiments. Differences between experimental groups were evaluated using one-way ANOVA followed by Tukey’s post hoc test. Statistically significant difference compared with the control: **—*p* < 0.01, *—*p* < 0.05.

The effects of *S. platensis* aqueous extract on fibroblast culture metabolic activity varied (Figure 3A): low concentrations of *S. platensis* (25 and 50 µg/mL) reduced it by 18 and 7%, respectively, compared to the control group; the 100 and 500 µg/mL of *S. platensis* extract increased fibroblast metabolic activity by 8%. The effect of the remaining tested concentrations on cell culture viability was similar to the control. These results indicate a nonlinear response, as there is no consistent trend of increasing or decreasing metabolic activity over the range of concentrations used in the study. C-PC at the concentrations 100– 250 µg/mL increased fibroblast PrestoBlue signal by about 8–14%, compared to the 100% control (Figure 3B). High concentrations of C-PC—300 and 500 µg/mL—did not affect fibroblast metabolic activity after 24 h.

In summary, the effects of S. *platensis* and C-PC depend on the type of skin cell culture and on the concentration of the preparation after 24 h incubation. Importantly, 100 µg/mL of *S. platensis* aqueous extract of and 200 µg/mL C-PC significantly reduced cellular metabolic activity in melanoma cells while enhancing it in keratinocytes and fibroblasts.

### 2.3. The Effect of Spirulina platensis Aqueous Extracts and C-Phycocyanin on the Wound Closure of Melanoma, Keratinocytes and Fibroblast Cells

**Figure 4:**
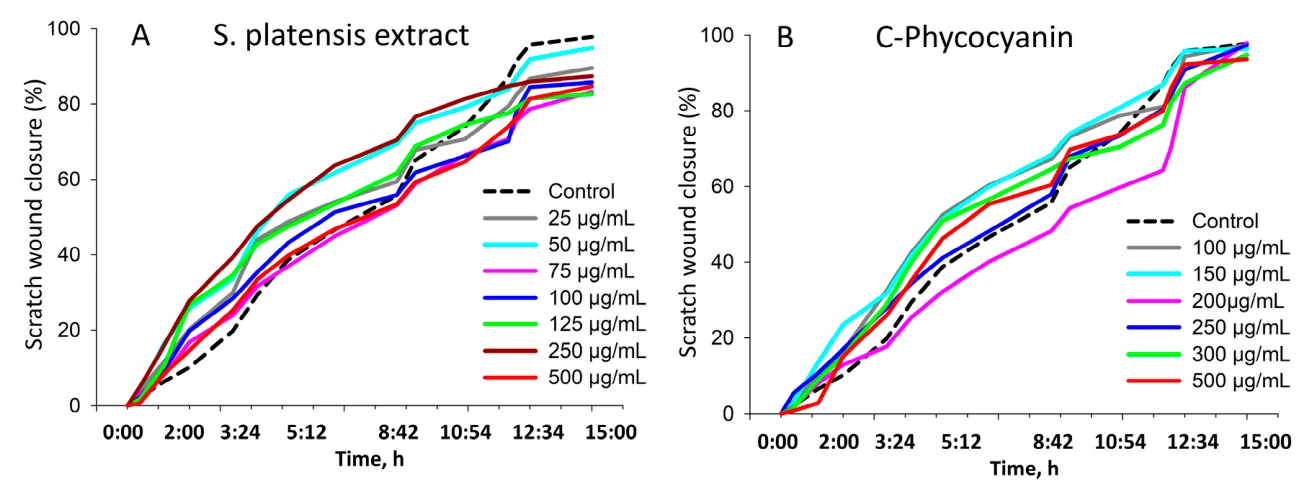
The effect of *Spirulina platensis* aqueous extract and C-Phycocyanin on the wound closure of melanoma cells. After treatment with different concentrations of *S. platensis* aqueous extract (25, 50, 75, 100, 125, 200, 250 and 500 µg/mL) or with C-PC solution (100, 150, 200, 250, 300, 500 µg/mL), the wound closure was assessed using an *in vitro* scratch assay and a Muvicyte microscope (PerkinElmer, Waltham, MA, USA). The observation was carried out for 15 h with melanoma cells. Repair of the Scratch in (**A**)—with *S. platensis* extract; (**B**)—with C-PC. The results of 3 independent experiments were analyzed using the Muvicyte Scratch Analysis software. Results are plotted as the repair rate of scratches in the cell line at a given time after the individual treatment period. Differences between experimental groups were evaluated using one-way ANOVA followed by Tukey’s post hoc test. Statistically significant difference compared with the control, *p* < 0.05.

We further examined the effects of *S. platensis* and C-PC on the wound healing of all three cell cultures using an *in vitro* scratch-wound-closure assay. The cells were exposed to the same concentrations of *S. platensis* aqueous extracts (25, 50, 75, 100, 125, 200, 250 and 500 µg/mL) or with C-PC solution (100, 150, 200, 250, 300, 500 µg/mL), a scratch (wound) was introduced into the confluent cell monolayer, followed by real-time imaging of the wells (see Materials and Methods, Section 4.5). The resulting images were analyzed using Muvicyte Scratch Analysis software, version 2.0.26 (PerkinElmer, Waltham, MA, USA). The repair time of the scratch plus extracts was compared with the control scratch repair per time unit and was expressed as a percentage. In melanoma, the total observation period was 15 h. We found that control melanoma cells closed the scratch by 97.75% during 15 h (Figure 4A) and it was considered 0%. We observed that the lowest concentrations of *S. platensis* extracts—25 and 50 µg/mL—reduced the rate of wound closure to a low degree, by 3% and 8% (both *p* < 0.05) over the 15 h period, if compared with the control, while 100, 125, 250 and 500 µg/mL of extracts slowed wound closure by about 12–14%, compared with the control (*p* < 0.05 for each concentration) after 15 h.

In melanoma cells, the C-PC concentrations of 100, 150, 200, 250 µg/mL, resulted in only a slight (2%) reduction in the wound-closure rate (*p* < 0.05) (the results were compared with a control of 97.75% within 15 h (Figure 4B). Similarly, higher concentrations, 300 and 500 µg/mL of C-PC, reduced the scratch-wound repair rate by 3% and 4% (*p* < 0.05), respectively, compared with the control, after 15 h.

**Figure 5:**
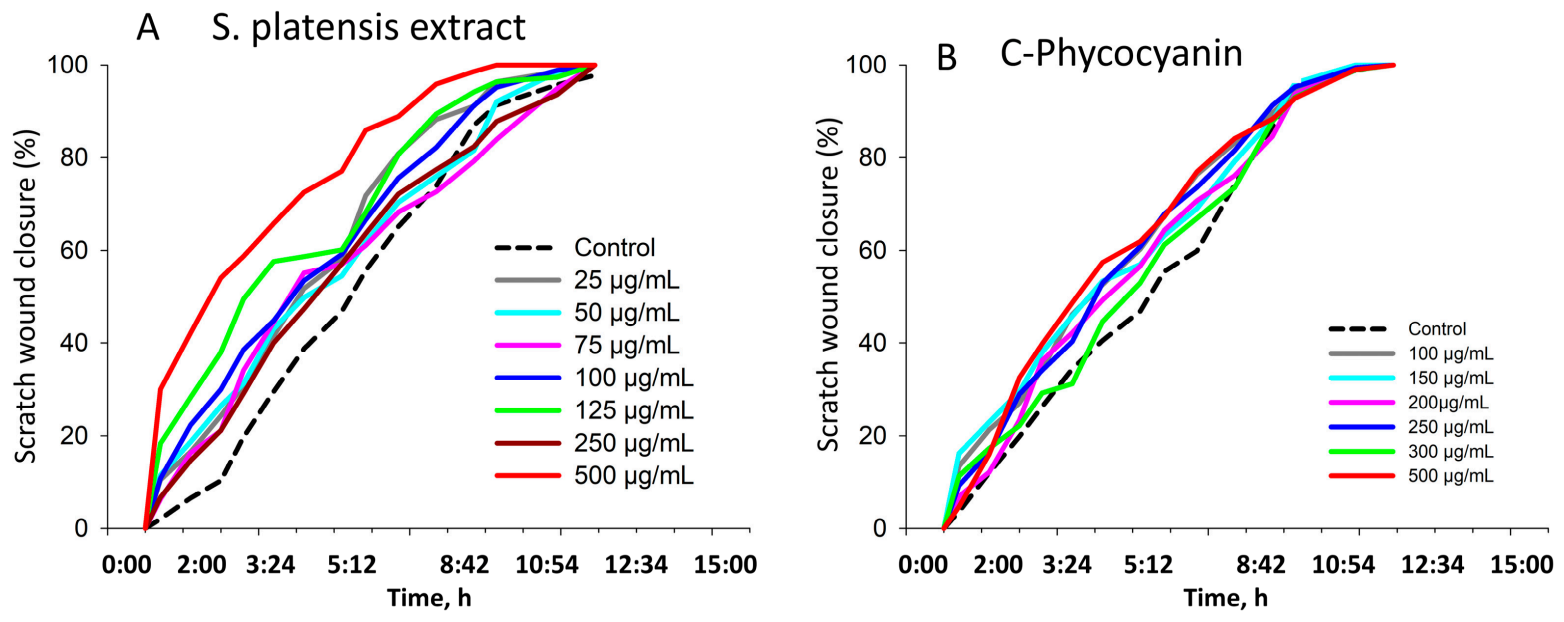
The effect of *Spirulina platensis* aqueous extract and C-Phycocyanin on the wound closure of keratinocyte cells. The concentrations of the preparations and the method are the same as described in Figure 4. The *in vitro* wound-closure observation of keratinocytes was carried out for 11 h and 54 min time period. Repair of the Scratch in (**A**)—with *S. platensis* extract; (**B**)—with C-PC. The results of 3 independent experiments were analyzed using the Muvicyte Scratch Analysis software. Results are plotted as the repair rate of scratches in the keratinocyte cell line at given time after the individual treatment period. Differences between experimental groups were evaluated using one-way ANOVA followed by Tukey’s post hoc test. Statistically significant difference compared with the control, *p* < 0.001; *p* < 0.01; *p* < 0.05.

Next, we conducted *in vitro* scratch-wound-closure experiments with *S. platensis* extracts on the keratinocyte cells (Figure 5A). Keratinocyte control cells reached 100% wound closure in 11 h and 54 min. Similarly, *S. platensis* extracts at 25, 50, 75, 100, 125, 200 and 250 µg/mL caused 100% wound closure after that time period. However, the highest extract concentration, 500 µg/mL, promoted complete scratch-wound closure within 7 h and 21 min (*p* < 0.001).

Next, we conducted *in vitro* scratch-wound-closure experiments with C-PC on the keratinocyte cells (Figure 5B). As all C-PC concentrations resulted in complete scratch closure, their effects were compared based on the time required to achieve complete closure. The fastest wound healing was observed at 100, 150 and 200 µg/mL concentrations: both 100 and 200 µg/mL C-PC exhibited similar activity within the same time period—in 9 h and 41 min (*p* < 0.05)—while 150 µg/mL accelerated this time to 8 h and 38 min (*p* < 0.01). A higher C-PC concentration, 250 µg/mL, resulted in 11 h and 5 min (*p* < 0.01). The highest C-PC concentrations, 300 and 500 µg/mL, did not affect the wound closure rate; it did not differ from the rate in the control group.

**Figure 6:**
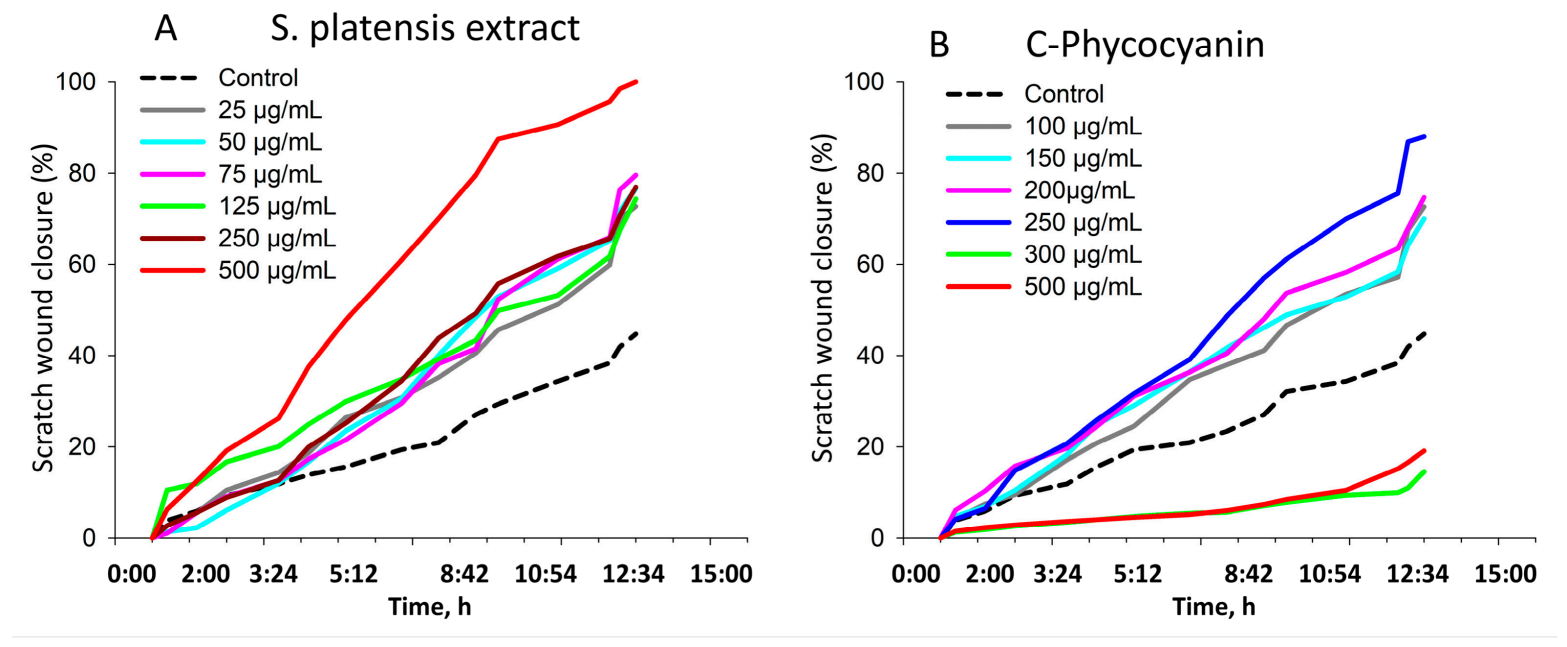
The effect of *Spirulina platensis* aqueous extract and C-Phycocyanin on the wound closure of fibroblast cell lines. The concentrations of the preparations and the method are the same as described in Figure 4. The observation was carried out for 13 h with fibroblasts. Repair of the scratch in (**A**)—with *S. platensis* extract; (**B**)—with C-PC. The results of 3 independent experiments were analyzed using the Muvicyte Scratch Analysis software. Results are plotted as the repair rate of scratches in cell lines at given time after the individual treatment period. Differences between experimental groups were evaluated using one-way ANOVA followed by Tukey’s post hoc test. Statistically significant difference compared with the control, *p* < 0.001; *p* < 0.01; *p* < 0.05.

The same concentrations of *S. platensis* aqueous extract were used to assess the wound closure rate of a fibroblast cell culture. This effect was observed over a 13 h period, during which the wound in the control cells closed by only 45% (Figure 6A). However, even a low concentration of the *S. platensis* extract, 25 µg/mL, accelerated the wound-closure rate by 28% (*p* < 0.001). Similarly, 50, 75, 125 and 250 µg/mL concentrations increased the scratch-wound closure by 32% (*p* < 0.001), 35% (*p* < 0.001), 30% (*p* < 0.05) and 32% (*p* < 0.001), respectively, after 13 h, compared with the control. It is important to note that the highest concentration of the *S. platensis* extract—500 µg/mL, was the most effective in promoting the closure of the fibroblast wound, with 100% (*p* < 0.05) after just 12 h. The results of the 100 µg/mL solution were statistically unreliable.

In fibroblast culture (Figure 6B), 100 and 150 µg/mL C-PC accelerated the wound closure by about 27% (both *p* < 0.05), compared with the control (the control wound closed by 45% in 13 h). A 200 µg/mL C-PC increased the wound-closure rate by 30% (*p* < 0.001), and the most effective concentration was 250 µg/mL, which accelerated the *in vitro* scratch-wound closure by 43% (*p* < 0.01). However, higher C-PC concentrations, 300 and 500 µg/mL, had the opposite effect and slowed wound healing by 30% and 25%, respectively (both *p* < 0.001).

Taken together, the *in vitro* scratch-wound-closure effects of the extracts and C-PC varied depending on both the skin cell type and concentration, indicating a cell-type- and concentration-dependent response.

### 2.4. The Antioxidant Activities of Spirulina platensis Aqueous Extract and C-Phycocyanin

Aqueous extract of *S. platensis* contains distinct bioactive compounds—such as C-PC, carbohydrates, free amino acids, phenolic acids, and flavonoids—that may contribute to its antioxidant properties [5]. In particular, phenolic acids and flavonoids exhibit strong antioxidant activity by scavenging free radicals and thereby reducing oxidative stress-induced cellular damage. We aimed to determine the antioxidant capacity of aqueous *S. platensis* extract and evaluate the relationship between the TPC and TFC of *S. platensis* and antioxidant capacity. Results showed that the antioxidant capacity of the aqueous extracts determined using the DPPH (2,2-diphenyl-1-picrylhydrazyl) radical scavenging method was 39.37 ± 1.33%. Similarly, the antioxidant capacity using the ABTS (2,2′-azino-bis-3-ethylbenzothiazoline-6-sulfanoic acid) radical scavenging method was 39.49 ± 1.25 µmol Trolox equivalent (TE)/g DW. The ability to reduce iron ions determined using the FIC (ferrous ion-chelating) method was 27.50 ± 1.23%. The statistical analysis revealed a significant correlation between the groups of total phenolic compounds, flavonoids and antioxidant capacity of the *S. platensis* aqueous extract (Table 2). The strongest Pearson’s correlation was observed between the total phenolics content and antioxidant capacity measured by the ABTS method (r = 0.918, *p* < 0.05), by DPPH (r = 0.896, *p* < 0.05) and by FIC (r = 0.886, *p* < 0.05). In addition, strong correlations were observed, although the correlation coefficients were lower between the total flavonoids and antioxidant capacity (Table 2). The results provide strong evidence to support the hypothesis that the examined parameters—TPC, TFC and antioxidant capacity—are interconnected and that *S. platensis* aqueous extracts explore protective effects against free radical production and for iron-chelation.

The antioxidant capacity of the C-PC determined using the ABTS radical scavenging method was 265.3 ± 24.5 µmol TE/g DW. The ability to reduce iron ions determined using the FIC method was 35.99 ± 0.5%. The DPPH radical scavenging method was not used to evaluate the antioxidant capacity of C-PPC due to spectral interference and the proteinaceous nature of the pigment. C-PC antioxidant capacity is important both as an isolated compound and as a major bioactive component of *Spirulina*, as it reflects its ability to scavenge reactive oxygen species and suggests potential relevance for protecting cells against oxidative stress.

## 3. Discussion

The present study demonstrated a concentration- and cell-type-dependent response of *Spirulina platensis* aqueous extract and C-PC on the cellular metabolic activity and wound healing of three distinct human skin cell lines: melanoma, keratinocytes, and fibroblasts. *S. platensis* extract concentrations ≥ 100 µg/mL effectively reduced cellular metabolic activity in melanoma cells while enhancing it in keratinocytes and fibroblasts after 24 h of incubation. Similarly, C-PC ≥ 200 µg/mL inhibited melanoma cellular metabolic activity and stimulated keratinocyte and fibroblast metabolic activity. Notably, both *S. platensis* aqueous extract and C-PC significantly accelerated wound closure in keratinocytes and fibroblasts but not in melanoma cultures, highlighting the characteristic response of the tested compounds between normal and malignant skin cells. Scratch-wound closure may reflect the combined contributions of cell migration and proliferation; therefore, the results are interpreted as enhanced *in vitro* scratch closure rather than direct evidence of increased cell migration.

In our study, the highest concentration of C-PC (500 µg/mL) exhibited a pronounced suppressive effect on melanoma cell metabolic activity. Therapeutically relevant *in vitro* concentrations of C-PC may range from 5 to 500 µg/mL [25], although concentrations up to 1000 µg/mL have been reported in studies investigating antimicrobial, cytotoxic, or pharmacological activities [26]. Differences in effective concentrations between studies may be attributed to variations in C-PC purity, treatment duration, cell type, and experimental design. The estimated C-PC content in the *S. platensis* extracts was relatively low compared with the concentrations tested in our experiments using C-PC itself (the corresponding C-PC concentrations in the *S. platensis* extracts using phosphate buffer ranged from 0.89 to 17.83 µg/mL). Therefore, the effects observed with the whole *Spirulina platensis* extracts cannot be attributed solely to C-PC, as other bioactive constituents may also contribute to the overall cellular response.

Our results are in agreement with previous reports demonstrating the anticancer activity of C-PC against various cancer cell types, including chronic myeloid leukemia [27], histiocytoma [28], cervical carcinoma [29], and melanoma cells [30,31]. C-PC has been shown to induce apoptosis, modulate intracellular oxidative status, and interfere with signaling pathways contributing to tumor progression, including GRB2/ERK and BRAF/MEK signaling in melanoma models. These observations support the hypothesis that the antiproliferative effect observed in melanoma cultures may result from similar biological processes. However, the precise mechanisms responsible for melanoma growth inhibition in our experimental model remain to be elucidated.

The present study was designed primarily to evaluate the functional effects of *Spirulina platensis* aqueous extract and C-PC on skin cell metabolic activity and wound healing, rather than to investigate their molecular mechanisms of action. Therefore, mechanistic endpoints such as ROS generation, apoptosis markers, and intracellular signaling pathways were not examined experimentally. The mechanistic interpretations discussed below are based on previously published evidence and are intended to support the biological interpretation of our findings rather than to provide direct mechanistic proof. Future studies should validate these pathways experimentally in melanoma cells, keratinocytes, and fibroblasts exposed to *S. platensis* aqueous extract and C-PC.

We found that *S. platensis* aqueous extract contained substantial amounts of C-PC, together with amino acids, phenolic compounds, flavonoids, and other bioactive constituents. Therefore, the biological activity of the aqueous extract likely reflects the combined action of multiple compounds rather than the effects of C-PC alone. This may partly explain why the responses to the aqueous extract and purified C-PC were not identical across all tested concentrations and cell types. The selective inhibition of melanoma cells accompanied by the stimulation of keratinocyte and fibroblast metabolic activity is particularly noteworthy, as such selectivity is desirable for dermatological and wound-healing applications.

To place our findings into a broader biological context, comparisons with previously published studies are warranted. Gunes et al. reported enhanced wound-healing activity and increased keratinocyte proliferation following treatment with topical formulations containing crude *Spirulina* extract [13], while Liu et al. demonstrated accelerated wound repair in a murine excisional wound model using *Spirulina*-derived proteins [15]. Similarly, Refai et al. showed that *Spirulina platensis* nanophytosome formulations improved wound healing, re-epithelialization, and collagen deposition in vivo [20]. Although these studies employed different formulations, extraction procedures, and biological models than those used in the present work, all consistently support a role for *Spirulina*-derived preparations in tissue repair and regeneration. Direct comparison between studies should nevertheless be interpreted with caution. The biological activity of *Spirulina* preparations depends strongly on the extraction methodology, resulting composition, concentration, and experimental model [13,20]. For example, crude aqueous extracts contain a complex mixture of phycobiliproteins, peptides, amino acids, carbohydrates, phenolic compounds, and minerals, whereas purified C-PC represents a single bioactive fraction [11,31,32]. Consequently, differences in cellular responses among studies may arise not only from concentration-dependent effects but also from substantial differences in chemical composition.

The distinct effects of *Spirulina platensis* extract on normal and malignant skin cells may be related to differences in redox regulation and metabolic status. Melanoma cells are characterized by elevated basal oxidative stress and altered redox homeostasis, making them more susceptible to biologically active compounds that influence intracellular oxidative balance [33]. Bioactive compounds present in *Spirulina*—particularly C-PC, phenolic compounds, and flavonoids—have been associated with antioxidant, anti-inflammatory, and antiproliferative activities in several experimental systems [29,31,32,34,35,36,37].

The observed stimulatory effects on keratinocytes and fibroblasts may be associated with the antioxidant properties of both preparations [38,39]. We demonstrated significant correlations between phenolic and flavonoid content and antioxidant capacity in the aqueous extract. Previous studies have also shown that C-PC protects skin cells from oxidative damage and ultraviolet-B-induced cellular injury [11]. In particular, Jang and Kim demonstrated protective effects of C-PC in HaCaT keratinocytes, whereas Kim et al. reported activation of the PKCα/βII-Nrf2/HO-1 pathway in human primary skin cells exposed to C-PC [32]. These findings are particularly relevant because they originate from skin-related cellular models that closely resemble the biological context of the present study. The enhanced *in vitro* scratch-wound closure observed in keratinocytes and fibroblasts may be associated with the cytoprotective properties of the extracts and their antioxidant capacity. However, chemical antioxidant assays do not fully reflect the complex antioxidant mechanisms operating in biological systems. Previous studies have suggested the involvement of MAPK/ERK- and PI3K/Akt-related pathways in tissue repair processes [31,32,40]. The lack of a clear concentration-dependent effect of the *Spirulina platensis* aqueous extract on fibroblast metabolic activity may be related to the complex composition of the extract and the potentially different effects of its bioactive constituents on cellular metabolism. It has been demonstrated that the effects of plant extracts and plant-derived bioactive compounds on fibroblast viability may vary depending on the type of extract or its concentration and do not always exhibit a clear concentration-dependent pattern [41,42].

*S. platensis* is recognized for its rich source of proteins, peptides, amino acids, vitamins, and minerals that can support cellular metabolism and tissue regeneration [43]. Together with the antioxidant and cytoprotective properties of C-PC, these characteristics may contribute to the beneficial effects observed in keratinocytes and fibroblasts. Importantly, the absence of comparable wound-healing stimulation in melanoma cultures further supports the biological selectivity of the investigated preparations.

Beyond their biological activity, both preparations possess formulation-related advantages that may facilitate future topical application. The results of this study indicate several advantages of *Spirulina platensis* aqueous extract and C-PC. The demonstrated antioxidant capacity may be relevant to the early stages of wound healing, as excessive oxidative stress can impair tissue repair and regeneration. Moreover, *S. platensis* aqueous extract contains more water-soluble compounds that might contribute to wound-closure processes; meanwhile, purified C-PC might help to standardize the preparation and control the dose. It is also important to point out that water-based preparations are more compatible with skin and do not cause irritation [44,45,46], which is more desirable for damaged skin regeneration and support the potential of these preparations for topical wound-healing formulations.

Collectively, these findings demonstrate that *Spirulina platensis* aqueous extract and C-PC exert concentration-dependent effects on metabolic activity and wound closure in normal and malignant skin cell cultures under *in vitro* conditions. The results are consistent with previous reports obtained using related skin-cell and wound-healing models; however, differences in extract composition, concentration ranges, and experimental systems should be considered when comparing studies. Taken together, these findings support the further investigation of *Spirulina platensis* aqueous extract and C-PC as naturally derived bioactive agents for skin regeneration. Future studies employing advanced mechanistic approaches, three-dimensional skin models, and in vivo validation are warranted to confirm their therapeutic value and support clinical translation.

## 4. Materials and Methods

### 4.1. Materials

Lyophilized powder of *Spirulina platensis* (100% Spirulina) was obtained from Bulk Powders™ (Pure Series, Colchester, UK; batch No. CO4 9QX). PrestoBlue reagent was from ThermoFisher Scientific (Waltham, MA, USA); aluminum chloride hexanitrate, acetic acid were from Carl-Roth (Karlsruhe, Germany). All other materials were purchased from Sigma-Aldrich (St. Louis, MO, USA), in between C—PC (C-Phycocyanin from *Spirulina* sp. lyophilized powder with 30.00–50.00% protein content).

Immortalized human keratinocyte cell line—the HaCaT keratinocyte cell line (RRID: CVCL_0038; Catalog No. 300493) and the human cutaneous melanoma cell line A375 (RRID: CVCL_0132; Catalog No. 300110)—were obtained from CLS Cell Lines Service GmbH (Eppelheim, Germany). The BJ-5ta human fibroblast cell line (RRID: CVCL_6573; Catalog No. CRL-4001) was obtained from ATCC (Manassas, VA, USA). All cell lines were obtained from commercial sources.

### 4.2. Preparation of Spirulina platensis Aqueous Extract and C-Phycocyanin Solution

An amount of 0.5 g of lyophilized *S. platensis* powder was dissolved in 10 mL of sterile physiological solution (pH of 6.5) and incubated for 24 h (at 4 °C). Then the samples were transferred to an ultrasonic bath (ELMASONIC P, Singen, Germany) for 30 min at 36 °C. After ultrasonication, the algae extracts were centrifuged at 8500× *g* 10 min (at 4 °C) (LMC-4200R, BioSan, Riga, Latvia) and the supernatants were filtered with a 0.2 µm pore size filter. The various *S. platensis* extract concentrations—25, 50, 75, 100, 125, 200, 250 and 500 µg/mL—were used for the experiments with skin cells.

The C-PC was dissolved in deionized water (1 mg/mL) and diluted with NaCl (0.9%); C-PC concentrations (100, 150, 200, 250, 300 and 500 µg/mL) were used in experiments with skin cells.

### 4.3. Cell Cultures Preparation

Cell culture procedures were carried out in a Class II biological safety cabinet (ESCO, Singapore). All three cell lines were stored in liquid nitrogen (−135 °C). A water bath and warm culture medium (DMEM (1X) + GlutaMAX-I, 10% Fetal bovine serum (FBS) and 1% penicillin and streptomycin antibiotic solution (10,000 IU/mL − 10,000 IU/mL)) were used to thaw the cells. The cell suspension was centrifuged for 3 min at 300× *g*. The supernatant was resuspended with 1 mL of culture medium. The resuspended cells were placed in a 75 cm^2^ cell culture flask and incubated at 37 °C and 5% CO_2_. After 24 h, the culture medium was replaced. When cells reached 70–80% confluence in the cell culture flask, they were subcultured. The growth medium in the flask was poured out and washed twice with phosphate-buffered saline (PBS), thus removing dead cells. Then 5 mL of trypsin was added to the flask and incubated for 5 min and were gently shaken to detach the cells from the bottom. The cell suspension was centrifuged for 3 min 300× *g*. The pellet was suspended with 1 mL of cell culture medium. Cells were seeded in a 96-well plate at the following densities: melanoma cells—2 × 10^3^, fibroblasts—3 × 10^3^, keratinocytes—3 × 10^3^, with 200 µL of growth medium and incubated for 24 h at 37 °C and 5% CO_2_ (Galaxy^®^ 170 S, New Brunswick™, Enfield, CT, USA). The next day, the culture medium was replaced with fresh culture medium (200 µL) with different concentrations of *S. platensis* or C-PC solutions and incubated for 24 h. After incubation, the measurements were performed.

### 4.4. Cellular Metabolic Activity Assay

Cellular metabolic activity was assessed using the reagent PrestoBlue. After 24 h exposure of cells to the *S. platensis* or C-PC solutions, 100 μL of PrestoBlue was added and incubated for 40 min at 37 °C. The fluorescence measurement was determined at 560 nm using a multifunctional microplate reader (Infinite 200 Pro M Nano Plex, Tecan Group Ltd., Männedorf, Switzerland). Cellular metabolic activity was normalized to the control group, which was set at 100%.

### 4.5. In Vitro Scratch-Wound Closure Assay

**Figure 7:**
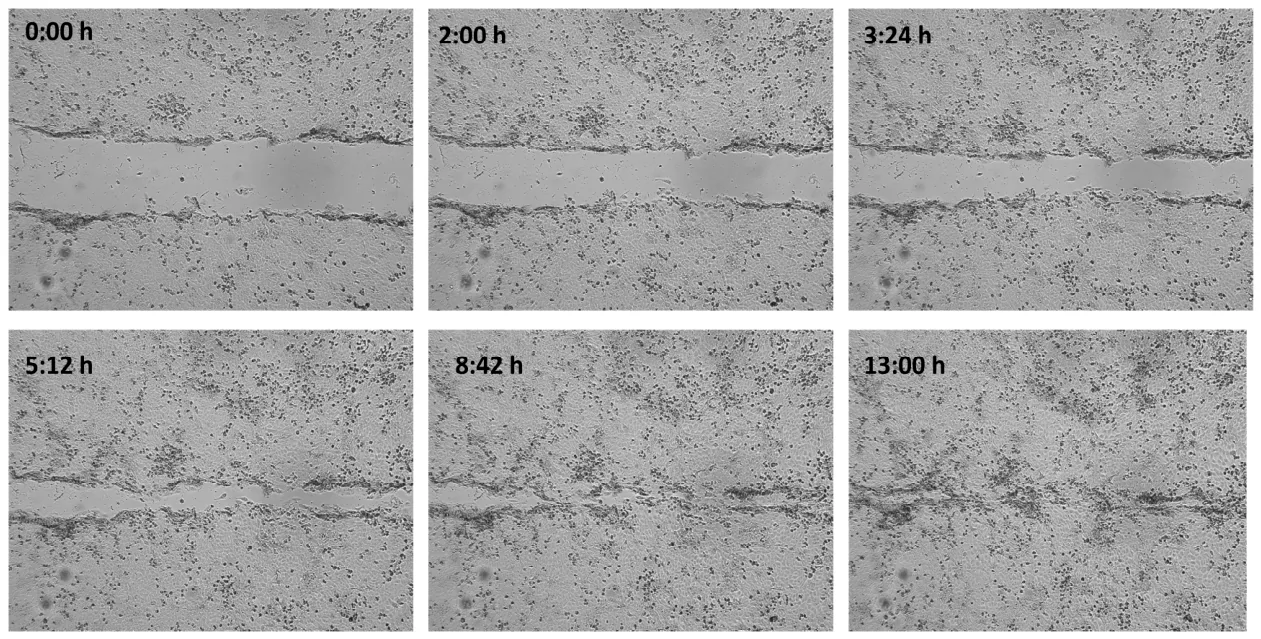
Representative images of the wound-closure model of fibroblast cell culture with the Muvicyte microscope (4× objective). Real-time images of the wells were taken at different time points. Images of a wound-closure assay experiment taken after different intervals of time.

Cell cultures were seeded in a 96-well slide plate with 200 μL of culture medium (DMEM (1X) + GlutaMAX-I, 10% FBS and 1% penicillin/streptomycin solution 10,000 IU/mL − 10,000 IU/mL) to reach 100% confluence. Then the culture medium was removed from the wells, washed with PBS and replaced with fresh culture medium with different concentrations of *S. platensis* extract and C-PC. The Muvicyte live-cell imaging system with integrated software and a handheld “Scratcher” device (Perkin Elmer, Waltham, MA, USA) was used to perform the wound-closure assay. The device was placed in an incubator and real-time images of the plates in the wells were taken. A “Scratcher” created the wound and the microscope (Leica DMi1, Leica Microsystems GmbH, Wetzlar, Germany) was used to check whether the scratches were uniform. The device takes a picture of the wound every 6 min until the wound closes (Figure 7), approximately for 13–15 h (the observation was carried out for 15 h with melanoma cells, 11 h and 54 min with keratinocytes and 13 h with fibroblasts). The results were analyzed using the Muvicyte Scratch Analysis software.

### 4.6. Determination of the Chemical Composition of Spirulina platensis Extract

#### 4.6.1. Determination of Amino Acids

Amino acid analysis was performed using an Acquity H-class (Waters, Milford, MA, USA) HPLC system coupled to a Xevo TQD (Waters, Milford, MA, USA) mass spectrometer. An amount of 1 μL of *S. platensis* extract was injected to a BEH Amide column (150 mm × 2.1 mm, 1.7 µm) at 25 °C. The mobile phase was delivered at a flow rate of 0.4 mL/min and consisted of 0.1% formic acid (eluent A) and acetonitrile (eluent B) using a gradient elution: 0–1 min—90% B; 1–6 min—70% B; 6–9 min—50% B; 9–10 min—the column was rinsed with 50% B; 10.1 min—the gradient was returned to its original composition; total analysis time—13 min. The operating conditions of the mass spectrometer were as follows: positive electrostatic spray ionization (ESI^+^) at 3.5 kV, cone voltage—30 V, desolvation gas flow—800 L/h, gas temperature—400 °C, ion source temperature—120 °C. Identification and peak assignment of amino acids in the *Spirulina* extract were performed by comparing their retention times and spectral data with those of standard compounds. Linear regression models obtained using the standard solution dilution method were used for quantitative analysis. The mass spectra of amino acids are provided in the Supplementary Materials, Figure S2.

#### 4.6.2. Determination of Organic Acids

Analysis of organic acids in *Spirulina* extract was performed with an Acquity H-class UPLC liquid chromatograph and a Xevo-TQD mass spectrometer. Chromatographic separation was performed with a Waters BEH C18 column (100 × 2.1 mm 1.7 µm). A constant temperature of 40 °C was maintained during the analysis. The eluents chosen were 0.1% formic acid solution (A) and acetonitrile (B). Gradient elution conditions are given in the table. Mass spectrometer parameters: negative electrospray ionization mode, solvent evaporation temperature 600 °C, gas flow 800 L/h. Ion source temperature—150 °C. The gradient elution conditions are summarized in Table 3.

The mass spectra of organic acids are provided in the Supplementary Materials, Figure S3.

#### 4.6.3. Determination of C-Phycocyanin Content

The content of C-phycocyanin in *Spirulina platensis* extract was determined using a spectrophotometric method [24]. Briefly, an appropriate amount of lyophilized biomass was suspended in distilled water or phosphate buffer (pH 6.8), filtered, and subjected to repeated freeze–thaw cycles to ensure efficient cell disruption and pigment extraction. The extract was then centrifuged at 9000× *g* for 5 min at 4 °C to remove cell debris and the supernatant was collected for analysis. The absorbance of the clear extract was measured at 620 nm and 652 nm using a UV–Vis spectrophotometer (Agilent Technologies, Santa Clara, CA, USA). The concentration of C-PC was calculated according to the following equation:

C-PC (mg/mL) = (A_620_ − 0.474 × A_652_)/5.34

The final C-PC content was expressed as mg/g DW. All measurements were performed in triplicate, and the results were expressed as mean ± SD.

#### 4.6.4. Determination of Total Phenolic Content

The total phenolic content (TPC) in *S. platensis* extracts was determined with a UV–Vis spectrophotometer (Agilent Technologies, Santa Clara, CA, USA) using the Folin–Ciocalteu method [47]. An amount of 1 mL (10 mg/mL) of the extract was mixed with 5 mL of 0.2 M Folin–Ciocalteu reagent. After 4 min, 4 mL of 7.5% (*w*/*v*) sodium carbonate solution was added, and incubated in the dark at room temperature (RT) for 60 min. The absorbance of *S. platensis* solutions was measured at a wavelength of 765 nm. The TPC was calculated according to the calibration curve (y = 6.6021x + 0.0634; R^2^ = 0.9983) and expressed in terms of milligrams of gallic acid equivalent (GAE) per gram of dry extract (mg GAE/g DW). Data are presented as mean ± SD based on three independent measurements.

#### 4.6.5. Determination of Total Flavonoid Content

The total flavonoid content (TFC) was determined by the aluminum chloride colorimetric method as described [46]. An amount of 0.2 mL *S. platensis* extract was mixed with 0.3 mL AlCl_3_ 10% (*w*/*v*), 2 mL of 96% (*v*/*v*) ethanol, 0.1 mL of 30% (*v*/*v*) acetic acid, and 0.4 mL of 5% (*w*/*v*) hexamethylenetetraamine solution. The entire mixture was diluted with purified water to 5 mL and kept in the dark at RT for 30 min. The reference solution was prepared in the same way, but without the extract added. The absorbance of the reaction mixture was measured at a wavelength of 407 nm. The TFC was calculated according to the calibration curve (y = 0.6749x + 0.0317; R^2^ = 0.9918) and expressed in milligrams of rutin equivalents (REs) per g of dry extract (mg RE/g DW). Data are presented as mean ± SD based on three independent measurements.

### 4.7. Determination of Antioxidant Capacity

#### 4.7.1. DPPH·Radical Scavenging Assay

The DPPH·(2,2-diphenyl-1-picrylhydrazyl) scavenging capacity was measured according to Yim and Nam (2016) [48]. An amount of 50 µL (10 mg/mL) of *S. platensis* extract was mixed with 2 mL of DPPH reagent. A blank sample was prepared by adding 2 mL of DPPH solution to 50 µL of purified water. The mixture was incubated for 25 min (until the reaction reached the steady state plateau) in the dark at RT. The decrease in absorbance due to the scavenging of DPPH was monitored spectrophotometrically at a wavelength of 515 nm. The radical scavenging capacity of *S. platensis* extract was calculated as the percentage of DPPH inhibition as follows:

DPPH scavenging capacity (%) = [(_Acontrol_ − A_sample_) ×100]/A_control_,

where A_control_ is the absorption of the blank sample and A_sample_ is the absorption of the solution with the analyzed extract. Data are presented as mean ± SD based on three independent measurements.

#### 4.7.2. ABTS·Radical Cation Decolorization Assay

An ABTS•+·(2,2′-azino-bis-(3-ethylbenzothiazoline-6-sulfanoic acid) radical cation decolorization assay was conducted according to the methodology as described by Yim and Nam (2016) [48]. The ABTS•+ radical cation was generated by reacting 50 mL of ABTS stock solution (2 mM) with 500 μL of 70 mM K_2_S_2_O_8_ solution; the mixture was allowed to stand in the dark at RT for 16 h before use. Before analysis, the ABTS•+ solution was diluted with purified water to obtain an absorbance at 734 nm. Subsequently, 30 μL (10 mg/mL) of *S. platensis* extract (or 1 mg/mL C-PC) was mixed with 3 mL of the ABTS•+ solution; the absorbance was measured at 734 nm after incubation for 30 min in the dark at RT.

The antiradical activity of the extract was based on the Trolox calibration curve (y = 0.0002x – 0.0013; R^2^ = 0.9902) and expressed as μmol Trolox equivalent (TE) per g DW. TE was calculated according to the following formula:

TE (μmol/g) = (C × V)/m,

where C is TE concentration of Trolox established from the calibration curve (in μM); V is the volume of the extract (in L); and m is the weight of the material (in g). Data are presented as mean ± SD based on three independent measurements.

#### 4.7.3. Fe^2+^ Chelating Efficacy

The ferrous ion-chelating (FIC) potential of *S. platensis* extract and C-PC was investigated spectrophotometrically by measuring the Fe(II)–ferrozine complex. To 1 mL (10 mg/mL) aliquot of the *S. platensis* extract (or 1 mg/mL C-PC), 50 μL of 2 mM FeCl_2_ solution was added. After 5 min, the reaction was initiated by the addition of 0.2 mL of 5 mM ferrozine solution. The mixture was vigorously shaken and allowed to stand at RT for 10 min. The absorbance of the solution was then measured at 562 nm. FIC capacity was calculated as a percentage using the following formula:

FIC (%) = [(A_control_ – A_sample_) ×100]/A_control_

where A_control_ is the absorbance of the reaction mixture with blank and A_sample_ is the absorbance of the reaction mixture with the plant extract. Data are presented as mean ± SD based on three independent measurements.

### 4.8. Statistical Analysis

Data are expressed as mean ± SD of 3–5 experiments on separate cultures or samples. Statistical comparison between experimental groups was performed using a one-way ANOVA followed by Tukey’s post hoc test using SPSS 20.0 software. *p* values < 0.05 were considered significant.

## Figures and Tables

**Table 1:** The amino acid content in *Spirulina platensis* extract.

Amino Acid	Concentration μg/g DW
Glutamate	116.84
Alanine	5.41
Serine	1.92
Glycine	0.13
Histidine	0.92
Leucine	0.46

**Table 2:** Correlation coefficients (r) between total phenolics, total flavonoids and antioxidant capacity of *S. platensis* aqueous extract.

Bioactive Compound Type	ABTS Assay (39.49 ± 1.25 µmol TE/g DW)	DPPH·Scavenging Assay (39.37 ± 1.33%)	FIC Assay (27.5 ± 1.23%)
Total phenolics content (7.89 ± 0.43 mg GAE/g DW)	0.918 *	0.896 *	0.886 *
Total flavonoid content (20.78 ± 0.98 mg RE/g DW)	0.710 *	0.858 *	0.695 *

* All experiments were performed in three independent experiments. The results are expressed as mean ± SD. Correlations between the investigated parameters were assessed using Pearson’s linear correlation coefficient. Differences between experimental groups were evaluated using one-way analysis of variance (ANOVA), followed by Tukey’s post hoc test. A *p* < 0.05 was considered statistically significant.

**Table 3:** Eluent flow rate and percentage of eluents A and B.

Time, Min	Eluent Flow Rate, mL/Min	Eluent A, %	Eluent B, %
0	0.4	95	5
0.5	0.4	95	5
3	0.4	65	35
5	0.4	40	60
7	0.4	10	90
8	0.4	5	95
8.1	0.4	95	5
10	0.4	95	5

## Data Availability

Data will be made available on request.

## Supplementary Materials

The following supporting information can be downloaded at: https://www.mdpi.com/article/10.3390/molecules31214020/s1, Figure S1: The effect of *Spirulina platensis* aqueous extract (A) and C-phycocyanin (C-PC) (B) on melanoma cell viability; Figure S2: Mass spectra of amino acids; Figure S3: Mass spectra of organic acids.

## Abbreviations

The following abbreviations are used in this manuscript:

ABTS	2,2′-azino-bis-3-ethylbenzothiazoline-6-sulfanoic acid
C-PC	Cyano-phycocyanin
DPPH	2,2-diphenyl-1-picrylhydrazyl
DW	Dry weight
GAE	Gallic acid equivalent
FIC	Ferrous ion-chelating
PBS	Phosphate-buffered saline
RT	Room temperature
RE	Rutin equivalent
TE	Trolox equivalent
TFC	Total flavonoid content
TPC	Total phenolics content
