# Supplementary material for: In Vitro Evaluation of Spirulina platensis Aqueous Extract and C-Phycocyanin on Skin Cell Metabolic Activity, Wound Healing and Antioxidant Activities
Source: Molecules. 2026 Nov 8;31(21):4020. doi: 10.3390/molecules31214020 (PMC13765470; doi:10.3390/molecules31214020)
Supplement: Supplementary file 1 [file molecules-31-04020-s001.zip › molecules-4641794-supplementary.pdf]

In vitro **evaluation of Spirulina platensis aqueous extract and C-phycocyanin on the skin cell metabolic activity, wound healing and antioxidant activities**

Gabija Nemeikšė, Jurga Bernatonienė, Asta Špadienė, Raimondas Benetis, Daiva Galinytė, Nijolė Savickienė, Gabrielė Balčiūnaitė-Murzienė, Aiste Jekabsone, and Ramune Morkuniene

Supplementary Materials

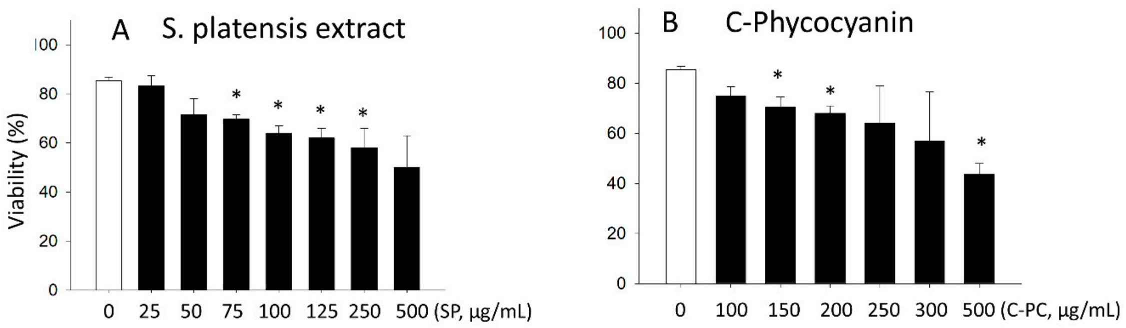

Figure S1. **The effect of *Spirulina platensis* aqueous extract (A) and C-PC (B) on the viability of melanoma cells.**

The viability of melanoma cell cultures was assessed by propidium iodide (PI) and Hoechst 33342 staining using a Muvicyte fluorescence microscope. The cells were seeded in a 96-well plate at 2 x 10^3 densities, with 200 µL of growth medium and incubated for 24 h at 37 °C and 5% CO_2. After 24 h, the cell medium was replaced with fresh medium and the cells were exposed to S. platensis (25, 50, 75, 100, 125, 200, 250 and 500 µg/mL) and C-PC (100, 150, 200, 250, 300, 500 µg/mL). After 24 h, cell cultures were incubated with a mixture of Hoechst 33342 (1.25 µg/mL) and PI (7 µM) for 10 min at room temperature, the plate was placed into the Muvicyte plate holder, and the "Attached Cell Counting" mode was used to capture images with blue/red fluorescence filters. Images were processed with the Muvicyte analysis software to quantify the number of viable and non-viable cells. The percentage of viable cells was calculated relative to the total number of cells per well. The data

*are presented as mean± SD of 3 independent experiments. Statistically significant difference compared with the control, * – p < 0.05.*

The effect of the *S. platensis* aqueous extract on cells was concentration-dependent, with the number of viable cells in the culture decreasing as the extract concentration increased after 24 h of treatment. *S. platensis* extract concentrations, 50 and 75 µg/mL, slightly reduced cell viability (Hoechst/PI), by 13% and 15%, respectively, compare with control (viability of control cells 85.3%). The more pronounced effect was observed at the higher extract concentration (125-250 µg/mL): the percentage of viable cells in culture reduced by 22-29%, whereas the lowest and highest concentrations did not produce statistically significant changes, compare with control (Figure S1A). C-PC solutions (150 and 200) after 24 h incubation reduced cell proliferation by 14% and 17%, compared to control (Figure S1B). The highest C-PC concentration – 500 µg/mL had a pronounced inhibitory effect and reduced viability by 46%. Others C-CP concentrations did not produce statistically significant effect on the melanoma viability after 24 h.

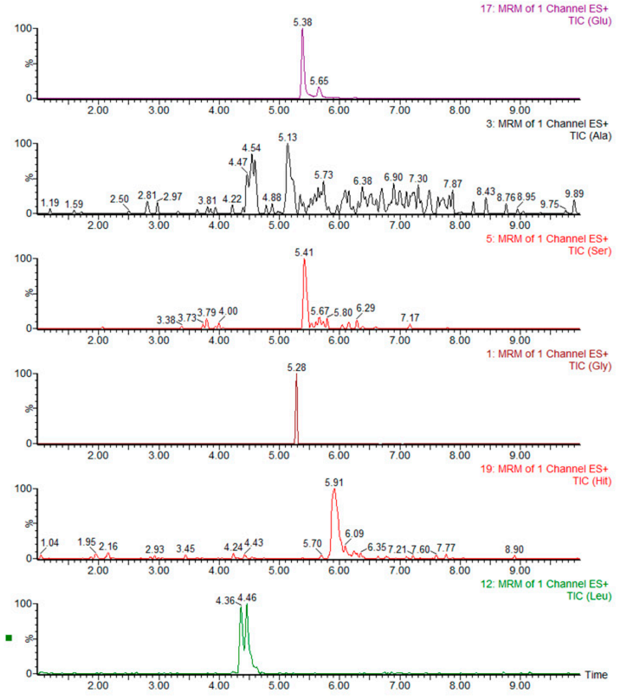

Figure S2. The mass spectra of amino acids.

Amino acid analysis was performed using an Acquity H-class (Waters, Milford, MA, USA) HPLC system coupled to a Xevo TQD (Waters, Milford, MA, USA) mass spectrometer as described in Materials and Methods 4.6.1. section.

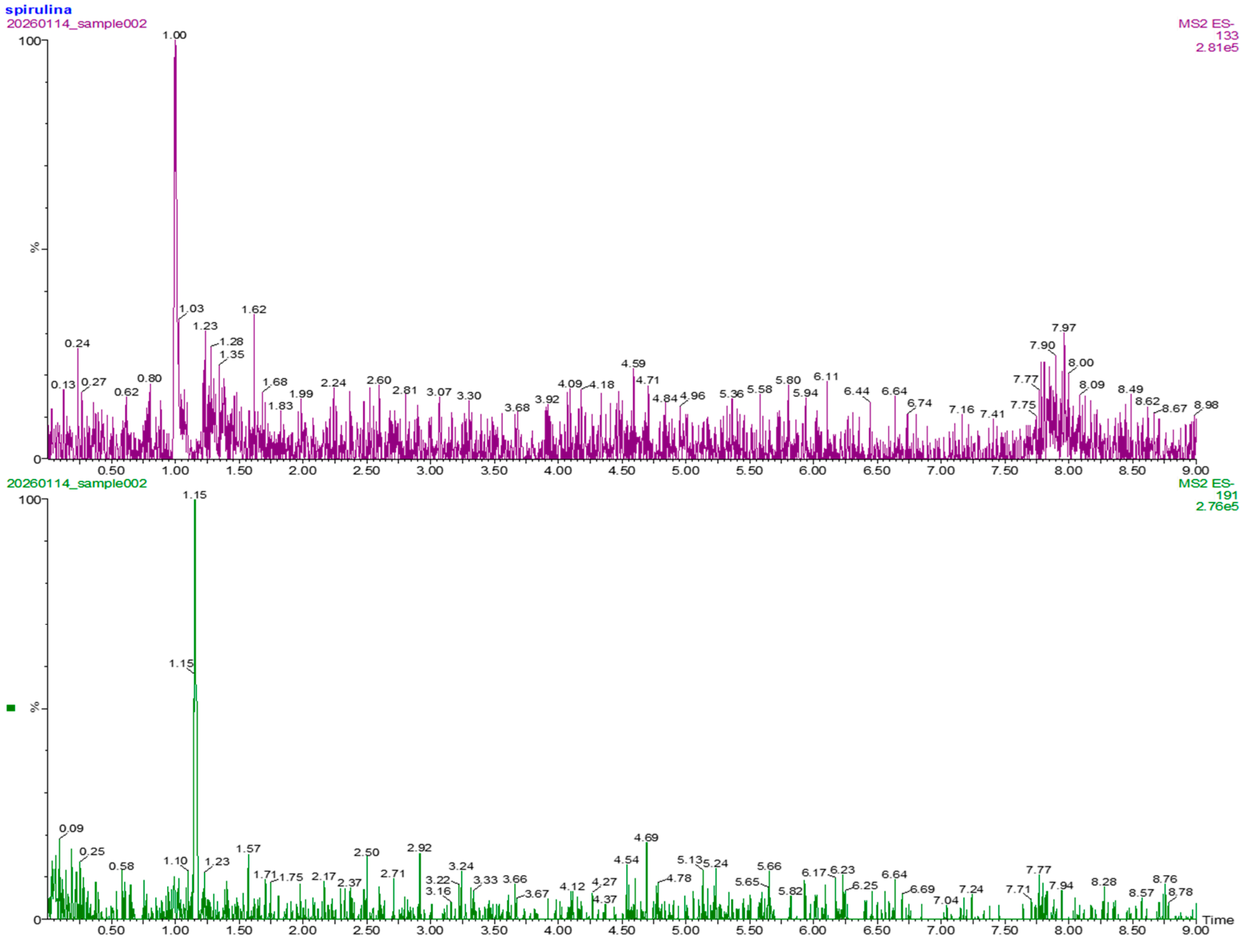

Figure S3. Mass spectra of organic acids.

Analysis of organic acids was performed with an Acquity H-class UPLC liquid chromatograph and a Xevo-TQD mass spectrometer as described in Materials and Methods 4.6.2. section.
